# Neuroprotective Copper Bis(thiosemicarbazonato) Complexes Promote Neurite Elongation

**DOI:** 10.1371/journal.pone.0090070

**Published:** 2014-02-28

**Authors:** Laura Bica, Jeffrey R. Liddell, Paul S. Donnelly, Clare Duncan, Aphrodite Caragounis, Irene Volitakis, Brett M. Paterson, Roberto Cappai, Alexandra Grubman, James Camakaris, Peter J. Crouch, Anthony R. White

**Affiliations:** 1 Department of Pathology, The University of Melbourne, Melbourne, Victoria, Australia; 2 Bio21 Molecular Science and Biotechnology Institute, Parkville, Victoria, Australia; 3 School of Chemistry, The University of Melbourne, Melbourne, Victoria, Australia; 4 Florey Institute of Neuroscience and Mental Health, Parkville, Victoria, Australia; 5 Department of Genetics, The University of Melbourne, Melbourne, Victoria, Australia; Nathan Kline Institute and New York University School of Medicine, United States of America

## Abstract

Abnormal biometal homeostasis is a central feature of many neurodegenerative disorders including Alzheimer's disease (AD), Parkinson's disease (PD), and motor neuron disease. Recent studies have shown that metal complexing compounds behaving as ionophores such as clioquinol and PBT2 have robust therapeutic activity in animal models of neurodegenerative disease; however, the mechanism of neuroprotective action remains unclear. These neuroprotective or neurogenerative processes may be related to the delivery or redistribution of biometals, such as copper and zinc, by metal ionophores. To investigate this further, we examined the effect of the bis(thiosemicarbazonato)-copper complex, Cu^II^(gtsm) on neuritogenesis and neurite elongation (neurogenerative outcomes) in PC12 neuronal-related cultures. We found that Cu^II^(gtsm) induced robust neurite elongation in PC12 cells when delivered at concentrations of 25 or 50 nM overnight. Analogous effects were observed with an alternative copper bis(thiosemicarbazonato) complex, Cu^II^(atsm), but at a higher concentration. Induction of neurite elongation by Cu^II^(gtsm) was restricted to neurites within the length range of 75–99 µm with a 2.3-fold increase in numbers of neurites in this length range with 50 nM Cu^II^(gtsm) treatment. The mechanism of neurogenerative action was investigated and revealed that Cu^II^(gtsm) inhibited cellular phosphatase activity. Treatment of cultures with 5 nM FK506 (calcineurin phosphatase inhibitor) resulted in analogous elongation of neurites compared to 50 nM Cu^II^(gtsm), suggesting a potential link between Cu^II^(gtsm)-mediated phosphatase inhibition and neurogenerative outcomes.

## Introduction

Altered biometal homeostasis is a central feature of many neurodegenerative disorders including Alzheimer's disease (AD), Parkinson's disease (PD) and motor neuron diseases. In AD, copper (Cu) and zinc (Zn) associate with extracellular amyloid-beta (Aβ) plaques, while intracellular Cu levels are decreased (Reviewed in [1] & [2]). As Cu is critical for many neuronal functions including synaptogenesis, normal synaptic activity and intracellular signaling [3], [4], targeting abnormal Cu homeostasis represents a potential therapeutic treatment for AD. Although a recent study found no cognitive improvements in AD patients receiving Cu supplements in their diet [5], early clinical trials of Cu/Zn ionophores including clioquinol (CQ) and PBT2 have demonstrated promising outcomes [6], [7].

Rather than acting as metal chelators to remove metal ions, the 8-hydroxyquinoline bidentate ligands, CQ and PBT2 can exert their effects in cell culture and animal models of AD by redistributing metal ions. CQ delivered Cu and Zn into cultured cells and stimulated Aβ degrading metallo-enzymes including matrix metalloprotease 2 (MMP2) [8]. PBT2 removed Zn from aggregated Aβ, redistributed the Zn into neuronal-like SH-SY5Y cells, inhibited glycogen synthase kinase 3 beta (GSK3β) and decreased calcineurin activity [9]. These effects may provide neuroprotective signaling changes in neurons.

Animal models of Aβ accumulation such as APP/PS1 and Tg2576 treated with CQ and PBT2 showed reductions in Aβ accumulation together with enhanced cognitive performance [10], [11]. However, it is not certain if these two observations are directly related. Our studies have provided evidence that metal delivery, or redistribution by ionophores such as CQ, PBT2 or metal complexes of bis(thiosemicarbazones) promote neuroprotective actions that are independent on changes to Aβ burden. Treatment of APP/PS1 mice with glyoxal-bis(N(4)-methylthiosemicarbazonato)-copper(II) (Cu^II^(gtsm)) resulted in enhanced cognitive performance compared to sham-treated animals without changes to Aβ deposition (although there was a correlation with decreased Aβ trimer levels) [12]. Malm *et al.* demonstrated that the metal ionophore pyrrolidine dithiocarbamate (PDTC) elevated brain Cu levels and improved cognition in APP/PS1 mice without altered Aβ levels [13]. We found that another bis(thiosemicarbazone) diacetyl-bis(N4-methylthiosemicarbazonato)-copper(II) (Cu^II^(atsm)), which is closely related to Cu^II^(gtsm), had a potent neuroprotective action in multiple models of Parkinson's disease and motor neuron disease, two diseases not associated with the Aβ peptide [14], [15]. Cu^II^(atsm) could also modify TAR-DNA binding protein 43 (TDP-43) metabolism, a protein central to neuropathology of amyotrophic lateral sclerosis and a sub-set of frontotemporal dementia cases [15], [16]. Combined, these studies provide strong evidence for a broad neuroprotective action of metal ionophores and Cu or Zn-complexes in models of neurodegeneration.

To further understand the neuroprotective action of metal ionophores *in vivo*, we studied dendritic spine density in a transgenic mouse model of Alzheimer's disease, Tg2576 mice, treated with PBT2 [17]. Dendritic spine density is a measure of synaptic strength, as dendritic spines are the site of interneuron synaptic connections in the brain. We observed a significant improvement in apical spine density in older Tg2576 mice treated with PBT2 [17]. A role for a general neurotherapeutic action by PBT2 in these mice was supported by elevation of several important neurotrophic proteins in the treated mice including pro-BDNF and TrkB. Cell culture studies demonstrated that PBT2 promoted neurite elongation[17]. This could be enhanced by the addition of Cu or Zn and prevented by co-incubation with the cell-impermeable Cu/Zn chelator, diAmsar [17]. These studies suggested that the neuroprotective action of PBT2 involved the redistribution of biometals such as Cu and/or Zn.

As Cu^II^(gtsm) and Cu^II^(atsm) have also demonstrated neuroprotective activity *in vivo* in murine models of neurodegeneration, we have now examined whether these metal-complexes induce similar changes in cultured cells. The metal complexes of bis(thiosemicarbazones) provide some advantages over 8-hydroxyquinoline compounds by being stable, pre-formed complexes that release Cu under specific intracellular environments [18]. This enabled us to determine if the delivery of Cu as a stable Cu^II^ complex results in beneficial action upon the intracellular release of the metal as Cu^I^. We investigated changes to total neurite numbers and neurite elongation (i.e., neurogenerative outcomes) in nerve growth factor-treated PC12 cell cultures. This neurosecretory cell line was chosen because it is the most well characterized model for neurite formation and growth, providing a useful model to examine how and where Cu complexes act. Cu^II^(gtsm) potently induced neurite elongation at concentrations as low as 25 nM. Treatment with Cu^II^(atsm), which is structurally comparable but does not readily release Cu inside cells under basal cellular redox conditions [19], required a 10-fold higher concentration to elicit a similar response. The potential mechanisms underlying the increased metal-associated neurite elongation were investigated. There was no specific association with kinase activation, but there was a significant association between inhibition of phosphatases and neurite elongation. Promotion of neurogenerative outcomes could potentially be a valuable *in vitro* biomarker for the neuroprotective action of these metal-complexes.

## Materials and Methods

### Materials

(Cu^II^(gtsm), Cu^II^(atsm), (gtsm)H_2_, diAmsar (1,8-diminosarcophagine) were synthesized as previously reported [20]–[23]. All other chemicals were purchased from Sigma-Aldrich (Castle Hill, NSW, Australia) unless specified otherwise.

### Pheochromocytoma (PC12) cell culture

PC12 cells (a kind gift from Dr Ann Turnley, Centre for Neuroscience, The University of Melbourne, Victoria, 3010, Australia) [17] were maintained in DMEM (Invitrogen, Mulgrave, Victoria, Australia) supplemented with 10% (v/v) foetal bovine serum, 5% (v/v) horse serum and 5 mL Penicillin/Streptomycin sulfate (solution contains 5000 units of penicillin and 5000 µg of streptomycin per mL, Invitrogen). Culture plates (Nunc, ThermoFischer Scientific, Scoresby, Victoria, Australia) were coated with 5 µg/cm^2^ collagen (Type I collagen, Rat tail derived, dissolved in 0.02 M acetic acid (Invitrogen)) for 1 hr at 37°C and then rinsed with PBS pH 7.4 before plating of cells, or stored at 4°C until used. For neurite elongation assays, cells were plated at low density (17×10^3^ cells/cm^2^) onto collagen-coated plates and allowed to settle for 24 hr. Medium was then replaced with serum-free medium containing nerve growth factor (NGF) (50 ng/mL (Invitrogen)) and cultured for a further 48 hr prior to treatments with metal-complexes (added into existing medium). Cells were examined for neurite elongation after a further 18 hr treatment. This protocol was employed to ensure that cells were at an early stage of neurite development to determine if this could be improved by treatment with Cu-complexes. Cells were not examined after longer periods because extensive neurite networks precluded quantitation of changes.

### Treatment of cells with metal-complexes

Metal-complexes were dissolved in dimethyl sulphoxide (DMSO) to achieve a 10 mM stock concentration. Dilutions were then performed where necessary so that the final volume of DMSO added to cells did not exceed 0.001% (v/v) of the final volume of culture medium in each well. Complexes were added to cells at indicated concentrations for 18 hr. Where indicated, inhibitors of extracellular signal regulated kinase (ERK) (PD98059, 10 µM), c-Jun N-terminal kinase (JNK) (SP600125, 10 µM), glycogen synthase kinase 3 (GSK3) (GSK3 inhibitor VII, 10 µM) and calcineurin (FK506, 5 and 10 nM) were added to cells for 18 hr. No evidence of significant metal-complex interaction with collagen matrix was found.

### Quantification of neurite formation and elongation

For assessment of neurite growth, cells were treated for 18 hr (after 48 hr NGF-differentiation) and fixed in 4% (v/v) paraformaldehyde (PFA) (ProSciTech, Thuringowa, QLD, Australia) in PBS (pH 7.4) for 20 min, then preserved in PBS containing 0.001% (w/v) sodium azide until bright field photographs were taken with a Leica inverted microscope with a Zeiss Axiocam digital camera at 500x magnification. ImageJ software (NIH) (with the neuronJ plugin) was used to quantify neuritogenesis and neurite elongation. Mean neurite length was calculated using NeuronJ by tracing the individual neurites of all the cells in 6 separate images taken randomly for each treatment (from three separate experiments). The length of each trace was measured, lengths were converted into µm and an average taken for each treatment. The proportion of cells with neurites 2x or longer than the width of the cell body was expressed as a percentage of the total. Neurite lengths were grouped into categories of 0–24, 25–49, 50–74, 75–99, 100–124 and greater than 125 µm and the mean number of neurites in each category was compared to assess if the distribution of neurite lengths was affected by treatment (n = 6 images from each of 3 separate cultures for each treatment). For assessment of neurite numbers and length, the investigator was blinded to the treatments.

### SDS-PAGE and Western Blot Analysis

After treatment, the media was discarded and the cells were scraped into Phosphosafe buffer (Phosphosafe Extraction Reagent, Merck, Kilsyth, VIC, Australia) supplemented with 100x phenylmethylsulfonyl fluoride (PMSF) and 50x deoxyribonuclease I (DNAse I, Roche Diagnostics, Castle Hill, NSW, Australia), using 50 µL for each well of a 6-well plate or 100 µL for a 100 mm dish. The cell suspension was then centrifuged at 16,000×g for 5 min. The cell pellet was discarded and an aliquot of the supernatant/lysate was taken for protein determination using the Pierce BCA protein determination assay as per instructions (Thermo Scientific, Rockford, IL, USA). Cell lysates were standardized using PBS (pH 7.4) and 4x sample buffer (250 mM Tris, 20% (v/v) glycerol, 8% (w/v) sodium dodecyl sulphate (SDS), 2% (w/v) β-mercaptoethanol, 0.01% (w/v) bromophenol blue) and incubated at 100°C for 5 min. Samples (10–20 µg) were then loaded onto 12% tris-glycine gels. Electrophoresis was performed at 125 V for 1 hr 45 min and proteins transferred onto PVDF membrane (Roche Diagnostics) at 25 V for 1 hr 45 min using transfer buffer (prepared from 25x stock (Invitrogen)) with 20% (v/v) methanol. PVDF membranes were blocked for 1 hr at room temperature with PBS (pH 7.4) supplemented with 0.05% (v/v) Tween-20 detergent (PBST) and 5% (w/v) skim milk powder. Membranes were incubated with primary antibodies all purchased from Cell Signaling Technology (Arunel, Queensland, Australia) and diluted at 1∶5000) overnight at 4°C in blocking solution, washed 3 times for 5 min with PBST, incubated for 2–3 hr at room temperature with secondary anti-rabbit antibody (diluted at 1∶500) in the blocking solution and then washed 4 times for 5 min with PBST before chemiluminescence imaging of protein bands using ECL Advance (GE Healthcare Biosciences, Castle Hill, NSW, Australia) and a Fujifilm LAS-3000 imager. Densitometric protein quantification was performed using ImageJ software and protein levels standardized relative to glyceraldehyde 3-phosphate dehydrogenase (GAPDH) expression.

### Immunofluorescence

After treatment with metallo-complexes, cells were rinsed once in PBS, fixed in 4% PFA in PBS for 20 min at room temperature and rinsed three times with PBS. Cells were permeabilized for 5 min with 0.01% (v/v) Triton-X dissolved in 10% (v/v) goat serum in PBS, followed by 3 washes with PBS and blocked in 10% (v/v) goat serum in PBS overnight at 4°C. Cells were washed 3 times with PBS and incubated with rabbit anti-phospho-JNK primary antibody (at 1∶500 dilution, Cat. # 4668, Cell Signaling Technology) in 10% (v/v) goat serum in PBS for a minimum of 2 hr at room temperature or overnight at 4°C. Cells were washed again 3 times with PBS and incubated with goat anti-rabbit secondary antibody (Alexafluor 488) at 1∶500 dilution in 10% (v/v) goat serum in PBS overnight at 4°C. Following three washes in PBS, cells were incubated for 5 min with 4′,6-diamidino-2-phenylindole dihydrochloride (DAPI) dissolved in PBS at 0.5 µg/mL. Cells were washed 4 times in PBS, mounted on coverslips with DAKO mounting medium (Dako, Campbellfield, VIC, Australia) and dried at room temperature.

### Inductively Coupled Mass Spectrometry

ICP-MS analysis of metal levels was performed as reported previously [24]. Cell pellets collected for metal analysis by ICP-MS were re-suspended in 50 µL of concentrated nitric acid (Aristar, BDH, Kilsyth, VIC, Australia) and left to digest overnight. Samples were heated for 20 min at 90°C to complete the digestion. The volume of each sample was reduced to approximately 40–50 µL after digestion, then 1 mL of 1% (v/v) nitric acid diluent was added to each cell sample. Measurements were made using an Agilent ICPMS 7700x series ICP-MS instrument under operating conditions suitable for routine multi-element analysis. The instrument was calibrated using 0, 5, 10, 50, 100 and 500 parts per billion (ppb) of certified multi-element ICP-MS standard calibration solutions (ICP-MS-CA12-1, ICP-MS-CAL-3 and ICP-MS-CAL-4, Accustandard) prepared in 1% (v/v) nitric acid for Cu, Fe, Zn and Mn. A certified internal standard solution containing 200 ppb of Yttrium (Y^89^) via T-piece was used as an internal control (ICP-MS- IS-MIX1-1, Accustandard). Results were expressed as micromole per liter concentrations of metal (µmol/L). The concentrations of Cu and other metals were calculated as µg of metal per mg of protein based on the protein concentration of the aliquot taken from each sample.

### Phosphatase assay

The SensoLyte FDP Protein Phosphatase Assay Kit Fluorimetric (Anaspec, Fremont, CA, USA) was used to quantify phosphatase activity. Following exposure to metal complexes, cells were rinsed twice in lysis buffer (supplied in kit) before scraping each sample into lysis buffer containing 0.2% (v/v) Triton-X as well as 10 µg/mL Aprotinin, 10 µg/mL Leupeptin, 100 µM PMSF and 10 µg/mL Pepstatin A (as instructed in assay kit protocol). The cell suspension was incubated at 4°C for 10 min with agitation and then centrifuged at 2,50×g for 10 min at 4°C. Supernatants were combined with the FDP-containing reaction solution (provided in the kit) in a black 96-well plate and fluorescence intensity was measured at Ex/Em = 490±20/520±20 nm using a WALLAC Victor^2^ plate reader (Perkin Elmer, Waltham, MA, USA). Data represented the mean ± standard error of the mean, n = 3 samples per treatment.

### Calcineurin assay

A colourimetric calcineurin assay was used to detect calcineurin activity (Merck KGaA, Darmstadt, Germany). Cells grown and treated in T75 flasks (Nunc) were scraped into Hank's balanced salt solution (HBSS, Invitrogen), centrifuged at 720×g for 3 min before resuspension in 1 mL of HBSS and standardized according to cell numbers (5×10^5^ cells/sample). Cells were pelleted by centrifugation at 720×g for 3 min before discarding the supernatant and lysing the cells with 100 µL (per sample) of calcineurin extraction buffer. The cell extracts were obtained by centrifuging the lysed cells at 16,000×g for 5 min. Resulting samples were desalted using spin columns (Corning, Tewksbury, MA, USA) containing Profinity^™^ IMAC uncharged resin (Bio-Rad Laboratories, Hercules, CA, USA). Assay reactions contained 25 µL of 2x assay buffer, 10 µL substrate and 15 µL of sample (or extraction buffer for negative control). The assay plate was incubated at 37°C for 30 min before termination of reactions by addition of 100 µL Malachite green reagent. A_620_ was measured after 20–30 minutes of colour development in a WALLAC Victor^2^ plate reader.

### MTT reduction and LDH assay

Cell health was determined using the 3-(4,5-dimethylthiazol-2-yl)-2,5-diphenyltetrazolium bromide (MTT) assay (Amresco Inc., Ohio, USA) and the lactate dehydrogenase (LDH) assay (Roche Diagnostics). Cells were grown and treated exactly as in the neurite elongation assay. After treatment of cells as described, an aliquot of cell media was removed, centrifuged to remove any cell debris, and stored for LDH assay. MTT assay was performed by addition of a 10% volume of 5 mg/mL MTT dissolved in PBS pH 7.4 to the existing media, resulting in a final concentration of 0.5 mg/mL. Cells were incubated at 37°C until blue crystals were visible by eye (approximately 30 min). Media was aspirated and replaced with 100 µL of DMSO per well to dissolve crystals. 100 µL aliquots were distributed into wells of a 96 well plate and A_570_ was measured in a WALLAC Victor^2^ plate reader. LDH assays were performed by addition of equal volumes (50 µL) of cell media aliquots and LDH reagent to each well of a 96 well plate. Plates were incubated at room temperature until development of a strong red colour. LDH release was measured by A_490–520_ using a WALLAC Victor^2^ plate reader. MTT reduction was calculated by subtracting the blank from all the readings and then calculating percentages of the untreated controls (controls had 100% MTT reduction). LDH release was similarly calculated by setting Triton X-treated controls at 100% release and converting the test values to a percentage of the Triton X control.

### Statistics

Data presented in graphs represents the mean ± standard error of the mean from analysis of phase contrast photomicroscopic images (n = 6 images from each of 3 separate cultures for each treatment). The investigator was blinded to the treatments. Densitometric analysis of western blots was performed using NIH ImageJ 1.43 software and adjusted according to loading control (GAPDH). Statistical analysis of results was performed using the two tailed student's t-test or ANOVA (one way or two way) with post hoc Bonferroni tests where appropriate. MTT and LDH data represented the mean ± standard error of the mean from the data calculated as a percentage of their respective controls (n = 6 wells for each treatment per experiment).

## Results

### Cu^II^(gtsm) increased cellular copper levels resulting in enhanced neurite elongation *in vitro*

NGF-treated PC12 cells were used to examine the effects of Cu^II^(gtsm) on total neurite numbers and neurite elongation (neurogenerative outcomes). Briefly, PC12 cells were treated with NGF for 48 hr and exposed to 50 nM Cu^II^(gtsm), Cu^II^(atsm), (gtsm)H_2_ (Cu-free ligand) or CuCl_2_, for 18 hr. Cells were then examined for: mean neurite length; mean number of neurites per cell; percentage of neurites that were at least twice (2x) the cell body width; and mean length of neurites when grouped according to length.

**Figure 1:**
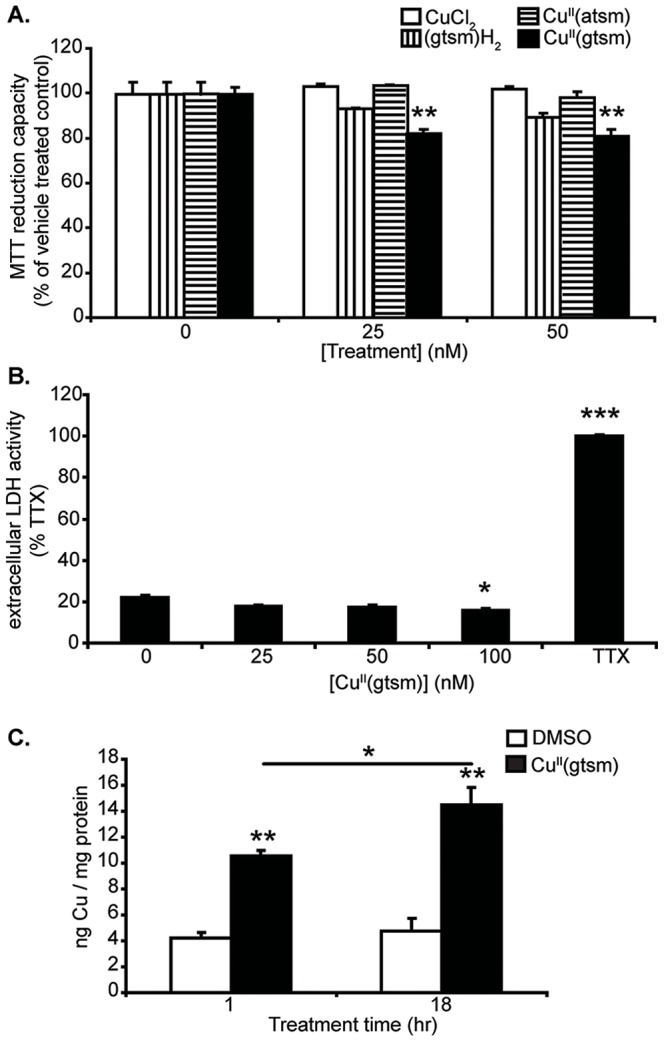
The effect of Cu^II^(gtsm) on MTT reduction, LDH release and Cu levels. The effects of CuCl_2_, (gtsm)H_2_, Cu^II^(atsm) and Cu^II^(gtsm) (25 and 50 nM) on NGF-treated PC12 cells was assessed. (**A**) The treatments used did not affect MTT reduction with the exception of 25 and 50 nM Cu^II^(gtsm) that inhibited MTT reduction slightly (n = 4/treatment). (**B**) LDH analysis of Cu^II^(gtsm)-treated cell cultures indicates that concentrations of up to 100 nM can be used with no significant increase in LDH release (n = 4/treatment). (**C**) ICP-MS results showed that 1 hr exposure to 50 nM Cu^II^(gtsm) increased cellular Cu content significantly and at 18 hr produced a significantly higher increase in Cu content than at 1 hr (n = 6/treatment). Values are mean ± SEM. *p<0.05, **p<0.01, ***p<0.001.

Metal accumulation and cell metabolism in response to treatments was first examined to determine the effect of Cu^II^(gtsm) on metal delivery and cell health. MTT assay results indicated that there was a small but significant loss of MTT reduction with 25 and 50 nM Cu^II^(gtsm) treatment (−17% and −18.5% respectively, with p<0.01 for both) (Figure 1A); however, an LDH assay showed that there was actually a small but significant decrease in LDH release with 100 nM Cu^II^(gtsm) (−6%, p<0.05) (Figure 1B). The reason for the slight differences between MTT and LDH assays is not certain. Changes to LDH assessments were not due to extracellular interactions between Cu and LDH (data not shown). It's possible that Cu^II^(gtsm) is able to induce some inhibition of cellular reductases without inducing cell damage. Whether there are some minor effects of Cu on lactate dehydrogenase export from cells is not known. However, the results demonstrate that no substantial changes to cells were found using these two common measures of cell health. No loss of MTT reduction was observed with the other compounds (Cu^II^(atsm), CuCl_2_ or (gtsm)H_2_) at the concentrations tested (Figure 1A). ICP-MS analysis of cells treated with Cu^II^(gtsm) (50 nM) indicated that cell-associated Cu increased significantly by 2.5-fold after 1 hr exposure and 3.1-fold after 18 hr exposure (p<0.001 for both) (Figure 1C).

**Figure 2:**
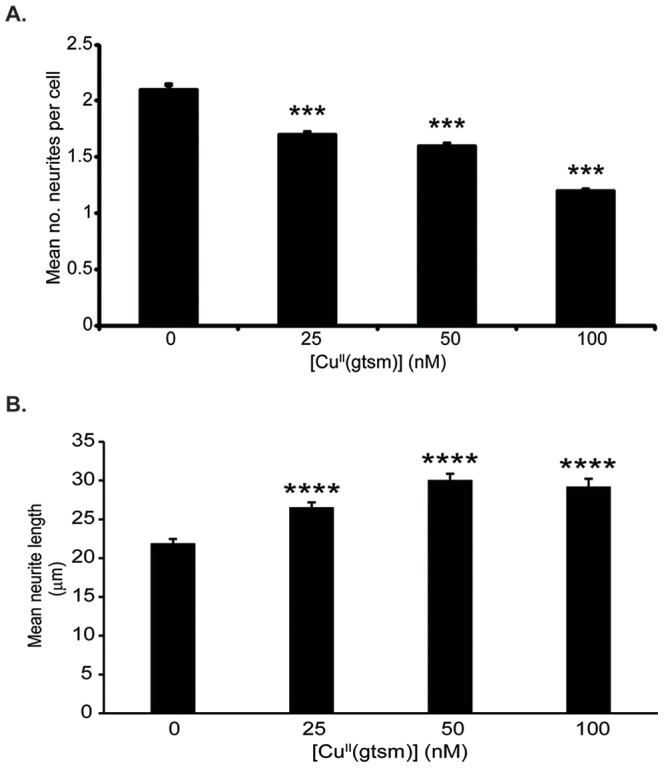
Cu^II^(gtsm) effects on neurogeneration in NGF-treated PC12 cells. The effect of Cu^II^(gtsm) (25, 50 and 100 nM, 18 hr) on total neurite numbers and neurite elongation was examined. (**A**) Cu^II^(gtsm) reduced the mean number of neurites per cell in a dose dependent manner (n = 3/treatment). (**B**) Cu^II^(gtsm) induced an increase in neurite elongation (assessed as mean neurite length) (n = 3/treatment). Values are mean ± SEM. **p<0.01, ***p<0.001.

**Figure 3:**
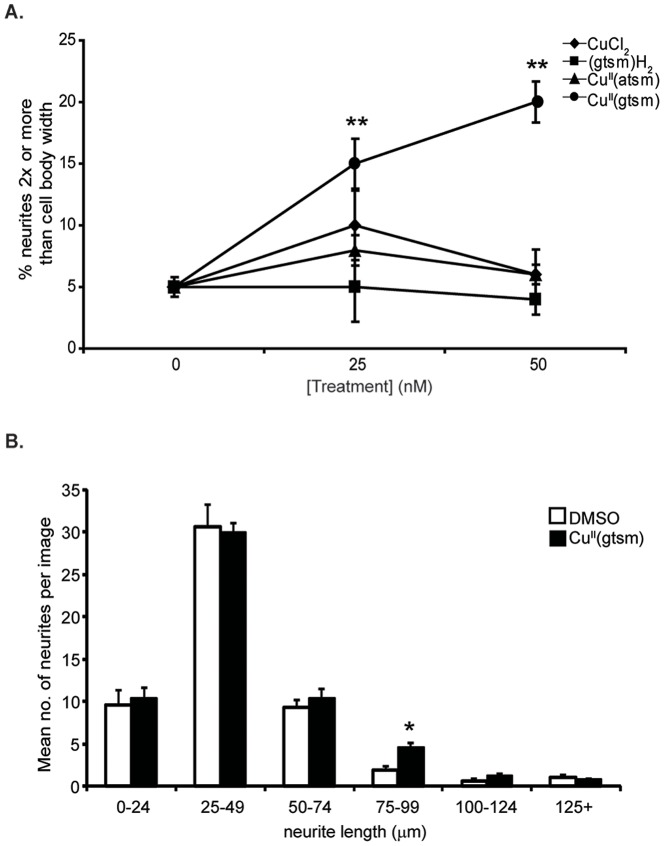
Cu^II^(gtsm) effects on neurite elongation of NGF-treated PC12 cells. (**A**) The effect of CuCl_2_, (gtsm)H_2_, Cu^II^(atsm) and Cu^II^(gtsm) (25 and 50 nM, 18 hr) on neurite elongation (assessed as % neurites 2x or more than cell body width). Cu^II^(gtsm) induced a significant increase in neurite elongation (at both concentrations used) whereas the other treatments had no effect (n = 3/treatment). (**B**) The effect of 50 nM Cu^II^(gtsm) on neurite elongation was examined further by grouping neurites according to length (measured in µm). Cu^II^(gtsm) induced a significant increase in the number of neurites in the 75–99 µm range. Values are mean ± SEM. *p<0.05, **p<0.01.

Assessment of the mean number of neurites per cell indicated that 25, 50 and 100 nM Cu^II^(gtsm) reduced neuritogenesis in a dose-dependent manner (−19%, −24% and −43% respectively, with p<0.001 for the three concentrations used) (Figure 2A). In contrast, Cu^II^(gtsm) exposure significantly increased mean neurite length at these concentrations (+21%, +37% and +34% respectively, p<0.0001 for the three concentrations used) (Figure 2B), as well as the percentage of neurites that were 2x or longer than the cell body width (+112% p<0.01, +75% p<0.01 and +63% p<0.05 respectively) (Figure 3A). The ability of Cu^II^(gstm) to induce increased neurite elongation was dependent on the delivery of Cu into cells, as treatment with the ligand alone (gtsm)H_2_, CuCl_2_ alone, or Cu^II^(atsm) (which does not readily release Cu under basal cellular conditions [19]), had no effect on neurite elongation at the concentrations tested (Figure 3A). Calculating the combined length of all neurites per cell revealed that Cu^II^(gtsm) had no significant effect on this parameter (data not shown). When neurites were grouped according to length, the number of neurites increased 2.3-fold (p, 0.01) in the size range of 75–99 µm while no change was observed in numbers of neurites in other size ranges (Figure 3B). This indicated that Cu^II^(gtsm) promoted elongation within a particular subset of neurites (based on length). Treatment of cultures with Cu^II^(gtsm) in the absence of NGF did not induce any neurite formation, consistent with the need for NGF to promote neurite outgrowth.

### Cu^II^(atsm) induced neurite elongation at higher concentrations than Cu^II^(gtsm)

**Figure 4:**
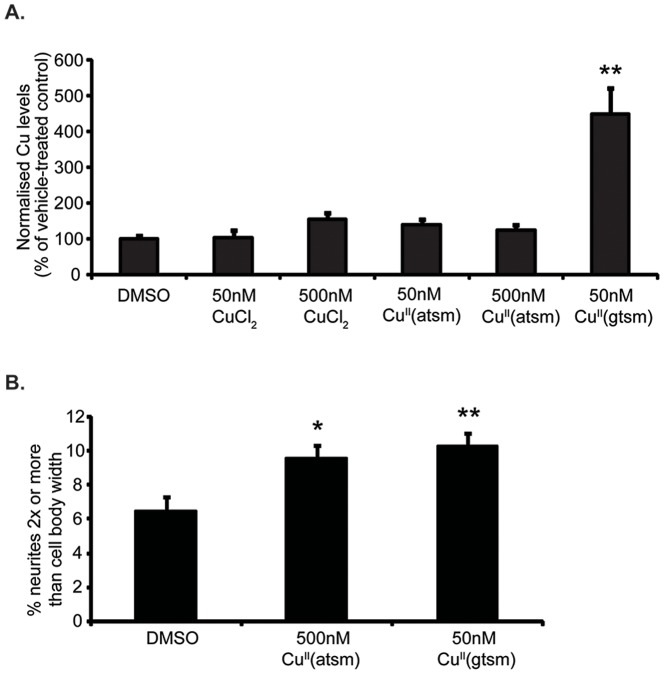
The effect of Cu^II^(atsm) on Cu levels and neurite elongation. (**A**) ICP-MS results showed that 18 hr exposure to 50 nM Cu^II^(atsm) increased cellular Cu content slightly while 500 nM had no effect on cellular Cu levels (n = 6/treatment). (**B**) The effect of Cu^II^(atsm) (500 nM, 18 hr) on neurite elongation (assessed as % neurites 2x or more than cell body width). Cu^II^(atsm) at 500 nM concentration induced a significant increase in neurite elongation similar to that induced by 50 nM Cu^II^(gtsm) (n = 4/treatment). Values are mean ± SEM. *p<0.05, **p<0.01.

To determine how the effects seen with Cu^II^(gtsm) related to Cu delivery, we analysed the ability of Cu^II^(atsm) to modulate neurite elongation. Cu^II^(atsm) is very similar in structure to Cu^II^(gtsm) with only two methyl groups differentiating it from Cu^II^(gtsm) [25]. We (and others) have shown that Cu is not readily released from Cu^II^(atsm) in most cells examined under basal conditions [19], [26]. Limited Cu release from Cu^II^(atsm) was supported here as treatment of PC12 cells with 50 and 500 nM Cu^II^(atsm) induced little change to cell-associated Cu when compared to 50 nM Cu^II^(gtsm) (Figure 4A), indicating that the Cu^II^(atsm) complex was mostly effluxed from the cell as previously reported [24]. When neurite elongation was examined, we found that although 50 nM Cu^II^(atsm) had no effect (Figure 3A), 500 nM Cu^II^(atsm) was able to induce a significant increase in neurite elongation (+48%, p<0.05) similar to that induced by 50 nM Cu^II^(gtsm) (Figure 4B). The ability of 500 nM Cu^II^(atsm) to induce similar neurite elongation to 50 nM Cu^II^(gtsm) but with little effect on Cu levels demonstrated that neurite elongation may not be directly related to Cu release by the Cu-complexes. Alternatively, the effects may be induced by significant alterations to specific sub-cellular pools or organellar localization of Cu that cannot be differentiated by ICP-MS and/or early transient changes to cellular Cu [24], [25], [27].

### Cu^II^(gtsm) inhibited kinases of the mitogen-activated protein kinase (MAPK) cell signaling pathway

**Figure 5:**
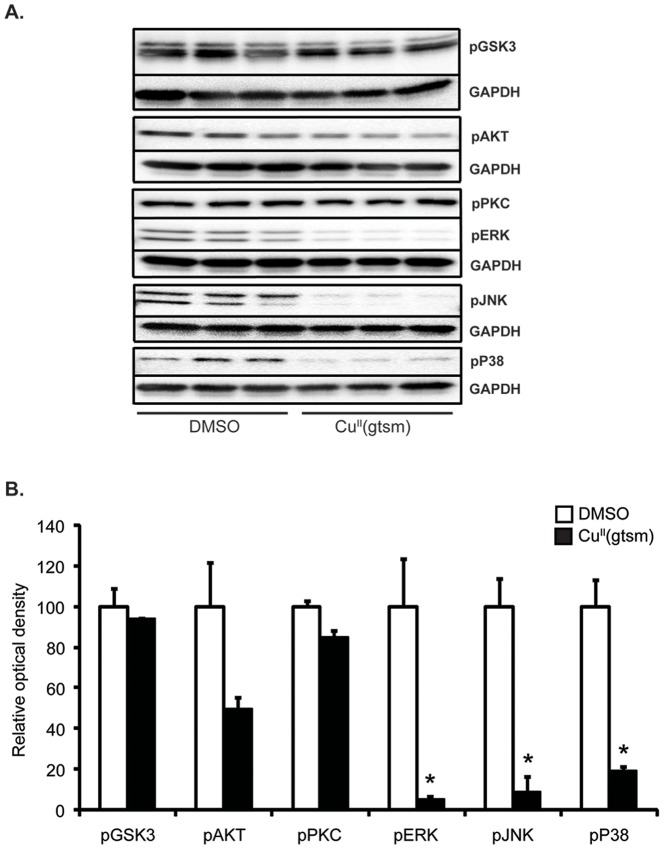
The effect of Cu^II^(gtsm) on kinase activation in NGF-treated PC12 cells. Activation of several cell signaling kinases was examined to determine which pathways might be involved in Cu^II^(gtsm)-mediated neurite elongation. (**A&B**) Cu^II^(gtsm) treatment decreased activation of ERK, JNK and p38 (n = 3/treatment). Values are mean ± SEM. *p<0.05

In order to examine the mechanism(s) by which Cu^II^(gtsm) modulated neurite elongation, the phosphorylation state of several key cell signaling kinases known to control neurite elongation was examined [28]–[30]. The basis for selecting these specific kinases was that Cu^II^(gtsm) modulated these kinases both in cultured cells and in brain tissue of treated mice in previous studies [12], [18], [19]. The kinase inhibitors did not significantly alter LDH release compared to Cu^II^(gtsm) treatment alone (Figure S1). Exposure of PC12 cells to Cu^II^(gtsm) (50 nM, 18 hr as for neurite analysis) significantly decreased activation of ERK, JNK and p38 (−87%, −85% and −64% respectively with p<0.05 for all three) but had no effect on glycogen synthase kinase 3β (GSK3β) or protein kinase C (PKC) (Figure 5A and B). Akt was decreased but not significantly. This was surprising as we have previously shown that Cu^II^(gtsm) can induce activation of these kinases, albeit at considerably higher doses [12], [31], although we have also reported that Cu^II^(atsm) can inhibit these kinases in SH-SY5Y cells [16].

**Figure 6:**
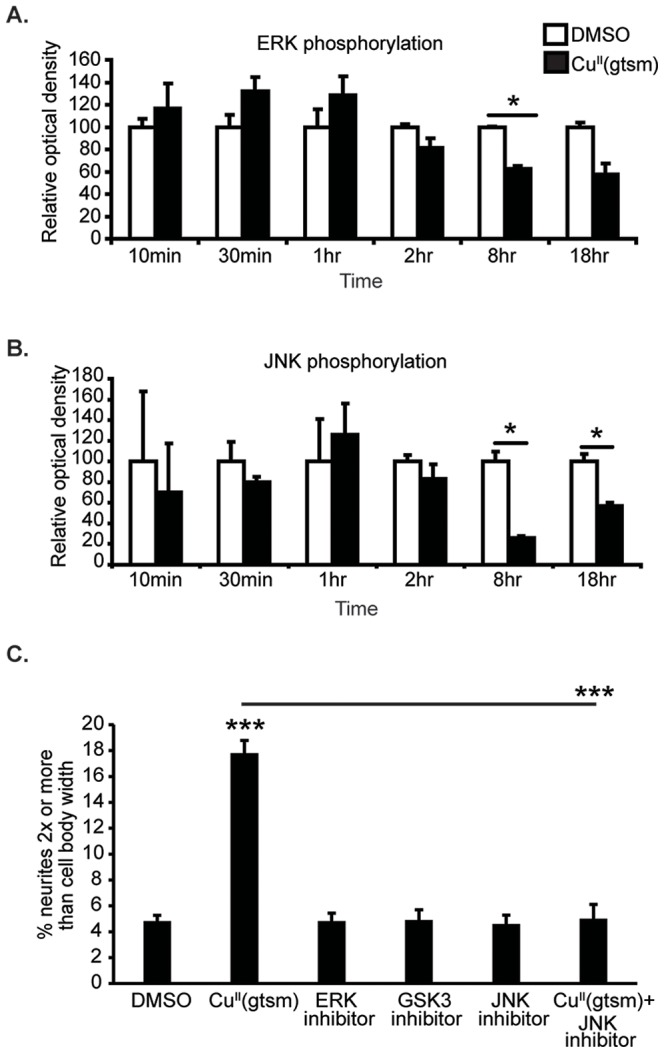
Timecourse of Cu^II^(gtsm)-mediated ERK and JNK phosphorylation and the effect of specific kinase inhibition on neurite elongation in NGF-treated PC12 cells. (**A & B**) Cu^II^(gtsm) (18 hr, 50 nM) decreased ERK and JNK activation from the 8 hr timepoint. (**C**) Neither JNK, ERK nor GSK3 inhibition had an effect on neurite elongation. Values are mean ± SEM. *p<0.05, ***p<0.001.

A time course analysis was then performed to determine if phosphorylation of ERK or JNK was affected at different time points up to 18 hr. While ERK activation showed a trend towards an increase within the first hour of treatment, this was not significant. ERK phosphorylation was significantly inhibited by Cu^II^(gtsm) after 8 hr (−40% p<0.05) (Figure 6A). Similarly, JNK activation was also inhibited after 8 hr and also 18 hr treatment with Cu^II^(gtsm) (−75% and −45% with p<0.05 for both time points) (Figure 6B). These findings suggest that altered cell signaling kinase activity by Cu^II^(gtsm) (i.e., from altered Cu levels associated with Cu^II^(gtsm) treatment) could be associated with the enhanced neurite elongation.

To test this, cells were treated with inhibitors of JNK (SP600125) 10 µM and ERK (PD98059) 10 µM for 18 hr to determine if inhibition of these kinases resulted in neurite elongation (these concentrations provide robust inhibition of JNK and ERK [32]). Neither inhibitor induced a significant change to neurite outgrowth, demonstrating that decreased JNK or ERK phosphorylation by Cu^II^(gtsm) was unlikely to be directly related to Cu^II^(gtsm)-mediated neurite elongation (Figure 6C). However to examine this further, we exposed cells to the JNK inhibitor (SP600125) in combination with Cu^II^(gtsm). Interestingly, SP600125 abrogated Cu^II^(gtsm)-mediated neurite outgrowth compared to Cu^II^(gtsm) alone (p<0.001) (Figure 6C), despite both compounds having analogous effects on JNK phosphorylation (inhibition of phosphorylation). The reason for this apparently conflicting result is not known. It is possible that SP600125 may target other kinases that are crucial to Cu^II^(gtsm)-mediated neurite elongation. However, examination of SP600125 action on casein kinase 1 (a known SP600125 target) did not reveal any inhibitory effect (data not shown), suggesting that the concentration used was unlikely to inhibit off-target kinases.

**Figure 7:**
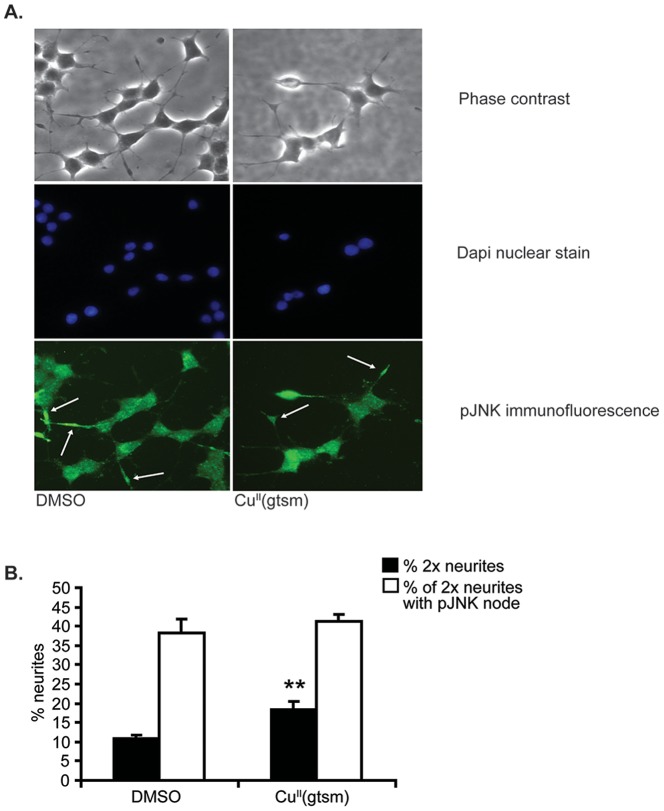
The localised effect of Cu^II^(gtsm) on JNK phosphorylation (pJNK) in NGF-treated PC12 cells. Cu^II^(gtsm)-mediated effect on pJNK was examined using immunofluorescence with rabbit anti-phospho-JNK primary antibody (Cat. # 4668, CST) and the percentage of longer neurites that have a pJNK node was quantified. (**A**) Images of pJNK immunofluorescence (**B**) The percentage of neurites that are two times or more than cell body width were increased by Cu^II^(gtsm) and the percentage of these longer neurites that have a pJNK node was found to be unchanged (n = 4/treatment). Values are mean ± SEM. **p<0.01.

An alternative hypothesis is that Cu^II^(gtsm) may have induced differential effects on JNK phosphorylation within the neurites and this could explain the neurite elongation observed with Cu^II^(gtsm) treatment. This possibility is supported by studies showing that JNK activity is required for neurite outgrowth [33], [34]. Examination of JNK phosphorylation by immunofluorescence was performed to observe localized changes in response to Cu^II^(gtsm). Immunofluorescence analysis demonstrated that neurites of longer length (2x cell body width) contained ‘nodes’ of phosphorylated JNK (Figure 7A). Quantification of the percentage of longer neurites containing these nodes revealed that there was no significant change in response to Cu^II^(gtsm) exposure (Figure 7B). This does not necessarily mean that phosphorylated JNK is not involved in neurite elongation, as basal levels could be required for neurite elongation to occur; however, Cu^II^(gtsm)-mediated elongation did not alter the formation of these phosphorylated JNK nodes.

### Cu^II^(gtsm) inhibited cellular phosphatase activity

**Figure 8:**
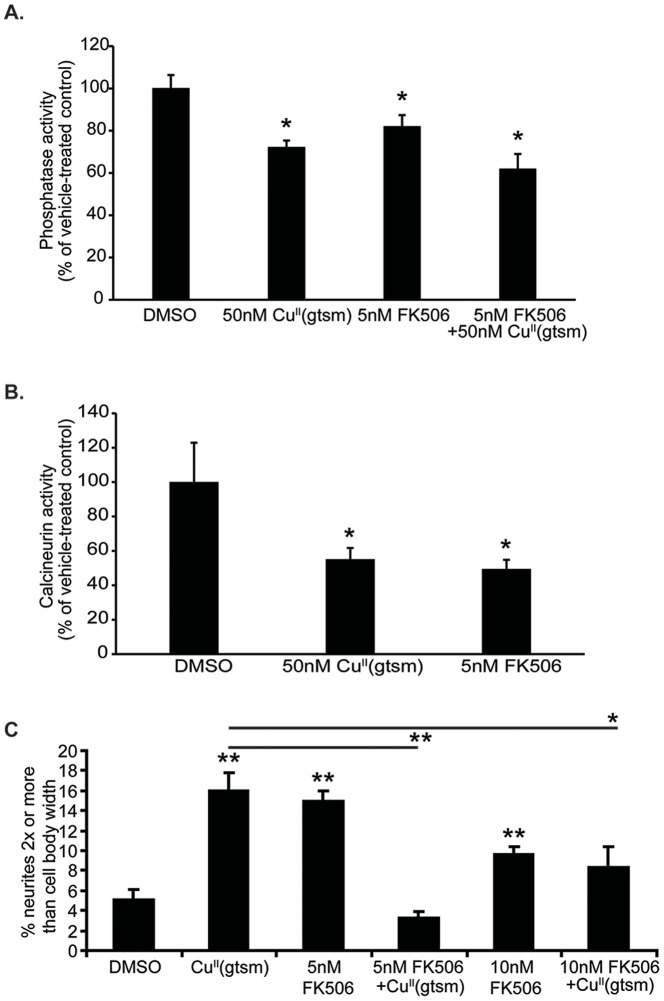
Effect of Cu^II^(gtsm) on phosphatase activity and effect on calcineurin. Following exposure to Cu^II^(gtsm) (50 nM, 18 hr) cells were assayed to determine overall phosphatase activity and also specifically for calcineurin activity. Cells were also exposed to the specific calcineurin inhibitor FK506 at 5 and 10 nM concentrations (18 hr) with and without Cu^II^(gtsm) (50 nM) and neurite elongation was assessed. (**A**) Cu^II^(gtsm) inhibited total phosphatase activity by 28% (n = 3/treatment). (**B**) Cu^II^(gtsm) inhibited calcineurin activity by 45% (n = /treatment). (**C**) Cu^II^(gtsm) and 5 nM FK506 each enhanced neurite elongation but when combined, their effects were blocked. At 10 nM concentration the FK506 effect on neurite elongation was not as strong, and again, when combined with Cu^II^(gtsm) the enhanced elongation was blocked (n = 4/treatment). Values are mean ± SEM. *p<0.05, **p<0.01.

As we observed no direct relationship between phosphorylation state of JNK or ERK and Cu^II^(gtsm)-mediated neurite elongation, we examined the relationship between phosphatase activity and neurite elongation in Cu^II^(gtsm)-treated cells. Phosphatases are the main effectors of kinase dephosphorylation and since Cu^II^(gtsm) inhibited several kinases we examined whether this was due to an up-regulation of phosphatase activity. As before, NGF-treated PC12 cells were exposed to Cu^II^(gtsm) (50 nM, 18 hr) and then assayed for broad spectrum phosphatase activity. Interestingly, Cu^II^(gtsm)-treated cells showed a 28% decrease in phosphatase activity (p<0.01) (Figure 8A). The phosphatase assay we used contained FDP, which is a highly sensitive fluorogenic substrate for most phosphatases, i.e., alkaline phosphatases, protein tyrosine phosphatases and serine/threonine phosphatases. Calcineurin inhibition by FK506 has previously been shown to enhance NGF-mediated neurite elongation *in vitro* [35] and improve Aβ-induced spine loss in APP/PS1 mice [36]. Cells were therefore assayed for calcineurin using a specific calcineurin (PP2B) activity assay. Cu^II^(gtsm) treatment was found to inhibit total cellular calcineurin activity by 45% when compared to controls (p<0.05) (Figure 8B). In contrast, no effect of Cu^II^(gtsm) was seen on protein tyrosine phosphatases at 50 nM (data not shown).

The inhibitory effects of Cu^II^(gtsm) on phosphatase activity were contrary to our initial expectation (due to the decreased phosphorylation of JNK and ERK). Therefore, we examined whether this inhibitory effect on phosphatase activity could be associated with neurite elongation. Since total cellular calcineurin activity was decreased by 45% in Cu^II^(gtsm)-treated cells, it is possible that localized changes to calcineurin e.g. in neurites, were responsible for the altered neurite elongation (it is not feasible to measure this using current calcineurin assays). Therefore, we examined the effect of treating cells with 5 nM calcineurin inhibitor, FK506 [37], [38]. FK506 (5 nM) did not affect cell LDH release when added alone or with Cu^II^(gtsm) (Figure S1B). This concentration of FK506 was found to decrease overall phosphatase activity by 18% and decreased calcineurin activity by 50% (Figure 8A and B). This treatment increased neurite elongation to a level equivalent to 50 nM Cu^II^(gtsm), supporting a potential relationship between phosphatase inhibition and neurite elongation (Figure 8C). We also examined the effect of 5 nM FK506 combined with Cu^II^(gtsm) as well as 10 nM FK506 with and without Cu^II^(gtsm). Alone, 10 nM FK506 enhanced neurite elongation but to lesser extent than the 5 nM treatment. When either 5 or 10 nM FK506 was combined with Cu^II^(gtsm) the effect on neurite elongation was abolished suggesting that there is an optimal level of calcineurin inhibition for this process. We also examined whether lower concentrations of Cu^II^(gtsm) and FK506 resulted in synergistic effects on neurite elongation. However, treatment of cells with 10 nM Cu^II^(gtsm) and 1 nM FK506 had no effect on neurite elongation. The lack of improved elongation with combined Cu^II^(gtsm) and FK506 was unlikely to be due to increased cellular toxicity as no change in LDH activity was observed (Figure S1B). The results suggest that interaction of these agents negates the positive effects of each alone but the mechanism is unknown.

We examined whether other inhibitors of Ser/Thr phosphatases (okadaic acid and phenylarsine oxide) also induced elongation. However, the concentrations that inhibited phosphatase activity to the level induced by Cu^II^(gtsm) treatment were also partially toxic and therefore compromised the neurite elongation assay. Our data indicates that calcineurin inhibition may be involved in Cu^II^(gtsm)-mediated neurogenerative effects.

## Discussion

8-hydroxyquinoline-based compounds such as CQ and PBT2 have established the therapeutic potential of metal-modulation for the treatment of AD and have led to testing the effectiveness of other Cu-complexing compounds, such as bis(thiosemicarbazones) [6], [10], [39]. Cu^II^(gtsm) is a compound that enables delivery of Cu across the blood-brain barrier, making it potentially bioavailable to neurons. Treatment of APP/PS1 mice with Cu^II^(gtsm) resulted in cognitive improvements accompanied by decreased soluble oligomeric Aβ levels [12]. However, whether the cognition-enhancing activity of Cu^II^(gtsm) is related to altered Aβ levels or alternative effects induced by modulation of Cu homeostasis is not known. Recent studies demonstrated that PBT2, an 8-hydroxyquinoline metal chelator, reduced loss of apical spine density in Tg2576 mice, and parallel cell culture studies suggested a link between these neuroprotective effects and enhanced neurite elongation, which was dependent on the presence of extracellular metals (Zn and/or Cu) [17]. However, these studies did not clearly establish whether the delivery of metals into cells as preformed metal-complexes is the driving factor in the beneficial outcomes observed in treated neurons.

In the present study we used the stable bis(thiosemicarbazonato) metal complex Cu^II^(gtsm) which allows tighter control over metal delivery. We examined the effects of Cu^II^(gtsm) on neurogeneration-related end points using a PC12 cell culture model of neurite formation and elongation. We determined that while Cu^II^(gtsm) reduced the total number of neurites per cell compared to control cultures, it enhanced neurite elongation in a dose-dependent manner and this effect was dependent on the delivery/bioavailability of Cu. Increased neurite length *in vivo* can be accompanied by a decrease in the total number of neurites as the cell shifts resources from *de novo* neurite formation to increased length of major projections [40]. The effects of Cu^II^(gtsm) could be related to interaction of the complex with cell surface receptors. However, we have reported analogous actions using the structurally unrelated 8-hydroxyquinoline compound, PBT2, suggesting that it is more likely the delivery of metal, than complex-receptor interactions, that trigger the effects on neurites, but this remains to be confirmed.

Cu^II^(gtsm) can inhibit the activity of several kinases known to be involved in neural differentiation and plasticity [41], but we found no evidence that this change was related to the effects of Cu^II^(gtsm) on neurite elongation. Despite this, Cu^II^(gtsm) could inhibit the activity of phosphatases with a significant inhibitory effect specifically on calcineurin. Furthermore, inhibition of calcineurin with FK506 mimicked the effect of Cu^II^(gtsm) on neurite elongation potentially implicating a role for calcineurin and/or additional phosphatases in the neurogenerative effects observed.

The NGF-treated PC12 cell line provides a valuable model for investigating neurogenerative effects of potential therapeutic agents for the treatment of AD [42], [43]. However, unlike the *in vivo* studies involving mouse models of AD treated with Cu^II^(gstm), these experiments were performed on non-transfected cells that did not overexpress the amyloid precursor protein (APP) and therefore Aβ. The fact that Cu^II^(gtsm) induced neurite elongation in these cells (albeit within a narrow length range of neurites) is consistent with a broad neuroprotective action of bis(thiosemicarbazonato) complexes in multiple animal models of neurodegeneration [12], [14], [15], rather than a specific effect on Aβ production. However, it is not clear how such a specific change in neurites (within the 75–99 µm length range) could equate to broad neuroprotection. Instead, these results should be taken as an *in vitro* marker of neurogenerative activity of the Cu-complex rather than an indicator of its cognition enhancing mechanism.

Neuronal differentiation and maturation *in vivo* involve axon pruning (reviewed in [44]); however, this occurs mainly during development. In the healthy mature brain, most plasticity events involve changes (retraction and sprouting in different locations) in dendritic spine morphology and are accompanied by synapse elimination and formation respectively [45]. In neurodegenerative diseases such as AD, PD and Huntington's disease there is significant axon degeneration [46]. Whether the neurite elongation-enhancing property of metal-complexes such as Cu^II^(gtsm) could be beneficial is not known.

A relationship between Cu bioavailability and neurite elongation was reported by Birkaya *et. al.* [47] who found that NGF-mediated differentiation of PC12 cells caused accumulation of Cu, while decreasing cellular Cu (using tetraethylene pentamine) impaired NGF-mediated neurite elongation. Our results are consistent with a role for bioavailable Cu in essential neurogenerative processes. Several studies have reported an inverse relationship between bioavailable Cu levels and cognition (reviewed [48]). Moreover, the Cu transporter ATP7a has a critical role in synaptogenesis as El Meskini *et al.* demonstrated that ATP7A expression levels are developmentally regulated and shifted from the cell bodies of developing neurons to the extending axons, peaking prior to synaptogenesis or during injury-stimulated neurogenesis [49]. These studies provide strong support for Cu (and ATP7A) as a mediator of neurogenerative pathways. Should bioavailable Cu in specific brain regions be diminished in AD, as reported in a recent meta-analysis [2], compounds such as Cu^II^(gtsm) have the potential to enhance Cu-associated neuronal functions.

The effects of Cu^II^(atsm) were also examined. Cu^II^(atsm) was selected because of its relative resistance to cellular reductants, resulting in decreased Cu release inside the cell. Although 50 nM Cu^II^(atsm) had no effect on neurite elongation, MTT reduction or cellular Cu content, the 500 nM Cu^II^(atsm) dose increased neurite elongation significantly. This did not correlate with apparent cellular Cu levels as they were unchanged at 18 hr. However, this does not take into account either dynamic changes to Cu levels over the preceding 18 hr, as supported by our previous studies [24], [25], [27] or altered sub-cellular Cu pools. Indeed, we have shown previously that uptake by M17 (human neuroblastoma cells) of fluorescently tagged Cu^II^(atsm) resulted in colocalisation with lysosomal and autophagic structures, as well as endoplasmic reticulum [24], [25], [27]. Interestingly, the analogous neurite elongation-enhancing effects of Cu^II^(atsm) and Cu^II^(gtsm) shown here provide support for potentially similar mechanisms of action for these compounds *in vivo* [14], [15].

MAPK signaling is associated with neurite formation and elongation [50]–[53]. We examined activation of several MAPKs that could potentially be involved in Cu^II^(gtsm)-mediated enhanced cognition. ERK, JNK and p38 were potently inhibited while GSK3 and PKC were unaffected. Akt was moderately changed. However, when ERK or JNK were inhibited by pharmacological inhibitors, there was no change in neurite elongation suggesting that these kinases were not mediating the observed effects.

We found that Cu^II^(gtsm) treatment resulted in diminished phosphatase activity (Figure 8B), and that the calcineurin-specific inhibitor FK506 mimicked the effect of Cu^II^(gtsm) on neurite elongation (Figure 8C). This suggested calcineurin could be involved in the observed neurogenerative effects of Cu^II^(gtsm). Calcineurin regulates neuronal plasticity [54], [55] and its inhibition improves synaptic function [56]. Furthermore, calcineurin is a therapeutic target for AD as its inhibition can ameliorate dendritic spine density deficits in a mouse model of AD [36]. The cellular mechanism of action of PBT2 possibly involved inhibition of calcineurin in a metal-dependent manner [9]. It is possible that there may be localized changes in calcineurin activity that are critical for neurite elongation. However, this would be difficult to determine as the calcineurin assays require large numbers of cells to measure activity and this technique could not be feasibly applied to neurites alone. Alternatively, calcineurin acts in concert with other phosphatases to modulate neurite elongation as supported by the 28% inhibition of broad spectrum phosphatases by Cu^II^(gtsm) in our assays (Figure 8A).

Whether the neurogenerative effects of Cu^II^(gtsm) are related specifically to Cu, Zn or both metals is difficult to decipher. An increase in cellular Cu may in fact result in increased Zn bioavailability via displacement of Zn ions that are bound to metallothionein (MT) as MT has a higher affinity for Cu than Zn [57]. This would explain why increasing Cu, by Cu^II^(gtsm) treatment or Zn by PBT2-Zn treatment, has the same inhibitory effect on calcineurin. Although a decrease in phosphatase activity should result in an increase in phosphorylation of various kinases, we did not see increased phosphorylation of ERK, JNK or GSK3. This suggests that the altered phosphatase activity may be compartmentalized and only affect a subset of kinases in Cu^II^(gtsm)-treated cells.

In summary, our study suggests that the Cu-delivery compounds, Cu^II^(gtsm) and Cu^II^(atsm) have the potential to enhance neurogenerative processes by increased metal bioavailability. This protective action could account for some of the neurotherapeutic effects of these complexes in animal models of neurodegeneration. Regardless, the results provide a valuable *in vitro* measure of neurogenic activity of this class of compound [14], [15]. Inhibition of calcineurin, and additional phosphatases, may be involved; however, the sub-cellular localization of this effect and the modulation of downstream kinases needs to be determined. Our studies demonstrated that low, physiological, concentrations of a Cu-complex (25–50 nM) can be beneficial and promote neurogenerative outcomes in neuron-related cells. This study provides valuable support for the therapeutic potential of Cu^II^(gtsm) and PBT2 in the treatment of AD and other disorders where metal dyshomeostasis is involved.

## Supporting Information

Figure S1 **The effect of kinase inhibitors and calcineurin inhibitor on LDH release.** The effects of (ERK inhibitor), SP600125 (JNK inhibitor), GSK3 inhibitor VII and calcineurin inhibitor (FK506) on NGF-treated PC12 cells was assessed. (**A**) LDH analysis of cell cultures treated with ERK, JNK or GSK3 inhibitors indicates that the 10 µM concentration can be used with no significant increase in LDH release (n = 5/treatment). (**B**) LDH analysis of cell cultures treated with 1, 5 or 10 nM FK506 demonstrated that these treatments had no effect on LDH release (n = 5/treatment). Values are mean ± SEM. *p<0.05, **p<0.01, ***p<0.001.
(TIF)
