# Supplementary figures and images for: Neuroprotective Copper Bis(thiosemicarbazonato) Complexes Promote Neurite Elongation
Source: PLoS One. 2014 Feb 28;9(2):e90070. doi: 10.1371/journal.pone.0090070 (PMC3938583; doi:10.1371/journal.pone.0090070)

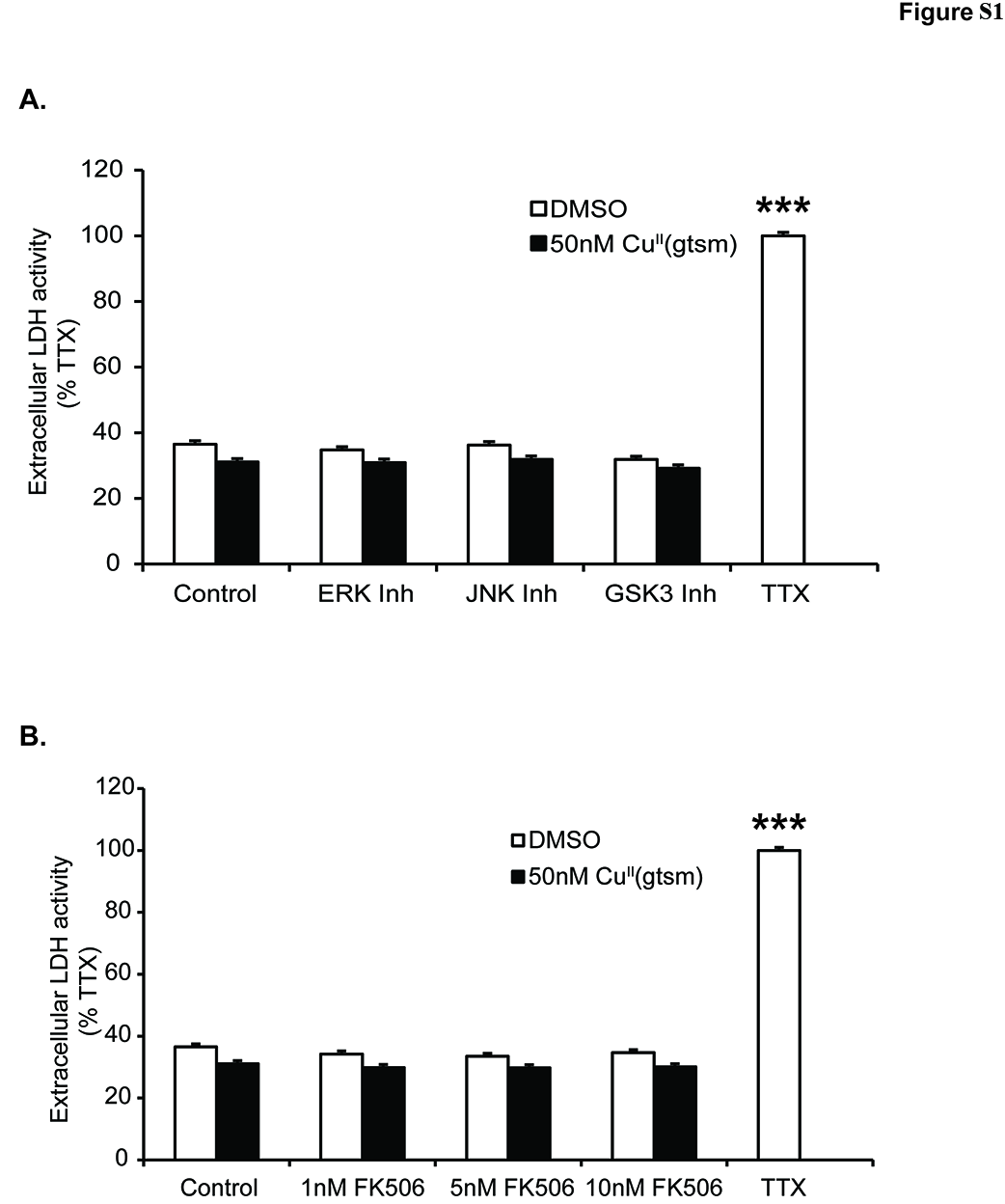

Supplement: Figure S1 — The effect of kinase inhibitors and calcineurin inhibitor on LDH release. The effects of (ERK inhibitor), SP600125 (JNK inhibitor), GSK3 inhibitor VII and calcineurin inhibitor (FK506) on NGF-treated PC12 cells was assessed. (A) LDH analysis of cell cultures treated with ERK, JNK or GSK3 inhibitors indicates that the 10 µM concentration can be used with no significant increase in LDH release (n = 5/treatment). (B) LDH analysis of cell cultures treated with 1, 5 or 10 nM FK506 demonstrated that these treatments had no effect on LDH release (n = 5/treatment). Values are mean ± SEM. *p<0.05, **p<0.01, ***p<0.001. (TIF) [file pone.0090070.s001.tif]
